# Unicompartmental Knee Arthroplasty Is Not Associated With Increased Revision Rates in Obese Patients

**DOI:** 10.1016/j.artd.2021.05.016

**Published:** 2021-06-23

**Authors:** Kevin F. Purcell, Benjamin M. Stronach, Marie Gene Almand, Doug Parsell, Trevor Pickering, R. Kerk Mehrle, Craig Winkler, Jeff D. Almand

**Affiliations:** a Department of Orthopaedic Surgery and Rehabilitation, University of Mississippi Medical Center, Jackson, MS, USA; b Mississippi Sports Medicine and Orthopaedic Center, Jackson, MS, USA

**Keywords:** Obesity, Unicompartmental, Knee, Arthroplasty, Revision, Complication

## Abstract

**Background:**

There is controversy among arthroplasty surgeons in regard to performing unicompartmental knee arthroplasty (UKA) in obese patients based on current literature. The aim of this study is to investigate whether UKA is associated with increased complications and revision rates in obese (body mass index [BMI] > 30 kg/m^2^), morbidly obese (BMI > 40 kg/m^2^), and super morbid obese (BMI > 50 kg/m^2^) patients.

**Methods:**

We retrospectively reviewed all UKAs performed at our institution from January 2008 to December 2017. A total of 2178 UKA procedures were performed during this period. The patients were categorized based on BMI to include normal weight (BMI = 20-30 kg/m^2^), obese (BMI ≥ 30.1-40 kg/m^2^), morbidly obese (BMI ≥ 40.1-50 kg/m^2^), and super morbid obese (BMI ≥ 50.1 kg/m^2^) groups. Record review was performed to obtain demographic data, need for revision (timing, type, and etiology), and complication rate and cause.

**Results:**

The 2178 UKA cases were eligible for inclusion in this investigation. We performed 2028 medial UKAs and 150 lateral UKAs. The mean clinical follow-up period was 3.7 years, and the mean time from index surgery to revision was 7.2 years. Of the 2178 UKA cases, 1167 had a 3-year minimum follow-up. The overall revision rate in all patients was 2.2%. There was no significant difference (*P* > .05) in revision rates among normal weight (3.0%), obese (2.7%), morbidly obese (1.9%), and super morbid obese patients (1.8%). Most failures in all groups were secondary to progression of osteoarthritis requiring total knee arthroplasty.

**Conclusions:**

Similar rates of revision were found for UKAs performed on obese, morbidly obese, or super morbid obese patients (≤2.0% revision rate) vs normal BMI (2.7% revision rate) patients. Progressive osteoarthritis was the most common mechanism of UKA failure. Obesity is not a contraindication for UKA despite previous recommendations to the contrary.

## Introduction

Obesity is an epidemic that continues to worsen within North American society with many deleterious health effects [1,2]. Obesity is a known risk factor for the development of osteoarthritis (OA), especially of the knee joint [[3], [4], [5], [6], [7], [8]]. As the rates of obesity continue to increase, we expect to see a concomitant increased demand for surgical intervention of the knee joint that includes high proximal tibial osteotomy, total knee arthroplasty (TKA), or unicompartmental knee arthroplasty (UKA).

There are multiple studies demonstrating increased complication rates in TKA on obese patients [[9], [10], [11], [12], [13], [14]]. There is an increased incidence of wound complications culminating in infection and increased overall rate of revision surgery [10,13,15]. Despite the increased potential for complication, surgical intervention affords these patients amelioration of pain and improved quality of life [10]. This creates a clinical and ethical dilemma for the arthroplasty surgeon.

Kozin and Scott published a hallmark article stipulating a set of criteria to determine the appropriate indications for UKA [16]. One criterion was the patient weight less than 82 kg (180 pounds). This recommendation in regard to weight to our knowledge, was based on their surgical experience, and was not necessarily driven by evidence-based medicine. This weight restriction was later increased to 90 kg [17].

There has been an increase in UKAs performed among arthroplasty surgeons for a myriad of reasons [18,19]. The benefits associated with performing UKAs are (1) reduced blood loss, (2) smaller surgical incision, (3) decreased hospital stay, (4) faster rehabilitation, (5) preserved biomechanics of the native knee, and (6) decreased infection rates [[20], [21], [22], [23]]. There is limited literature investigating UKAs in the morbidly obese (BMI ≥ 40 kg/m^2^) and the super morbid obese (BMI ≥ 50 kg/m^2^) population [24,25]. In addition, there is controversy among UKA literature about expanding UKA indications to obese or morbidly obese patients [[24], [25], [26]].

At our institution, BMI alone is not a contraindication for UKA, and patients are considered candidates for UKA with a BMI > 40 kg/m^2^ if other risk factors are controlled. Anecdotally, we have not experienced an increased complication profile in this patient population. The aim of this investigation is to determine if the complication and revision rate of UKA in obese population is increased in comparison to normal weight patients.

## Material and methods

This investigation was a retrospective chart review. After obtaining approval from our institutional review board, a database search was performed from January 2008 to December 2017 to identify all patients that underwent UKA. A total of 2178 UKA cases were performed during this period. Four fellowship-trained high-volume UKA surgeons performed all the surgeries.

### Inclusion criteria

Inclusion criteria were standardized to allow appropriate comparison to previous studies and include (1) isolated medial or lateral unicompartmental replacement; (2) no extensive involvement (>10%) of contralateral compartment or patellofemoral joint; (3) intraoperative assessment excluding any anterior cruciate ligament injury; (4) did not have any rheumatologic/inflammatory arthropathy; and (5) no prior knee procedures such as high proximal tibial osteotomy. All patients received the same fixed bearing UKA implant (Sigma HP Partial Knee; DePuy Synthes, Raynham, MA). The same implant was used to mitigate any possibility of confounding. All implants were cemented, and the tibial component was modular with a baseplate and polyethylene component. Patients must have failed a trial of conservative management before surgical intervention. Computerized knee navigation (Brainlab AG, Munich, Germany) was used for each UKA performed. Patients were excluded from this study if they did not receive the same implant, did not have BMI recorded in chart, or if their primary UKA was performed at an outside hospital.

### Chart review

A thorough chart review was performed to obtain demographic data for each patient. The information extracted was gender, age at time of surgery, laterality of procedure, tobacco usage, medial or lateral UKA, other comorbidities, BMI at the time of index procedure, and follow-up period. We recorded if there were any secondary procedures after index UKA. Failure was defined as conversion to TKA or revision of the femoral or tibial component. Polyethylene exchange was not considered a failure. Revision was defined as any surgery warranting implantation of new hardware excluding polyethylene. Complications were defined as any morbidity that necessitated a revision surgery. We divided these secondary procedures into conversion to TKA or revision of femoral/tibial components of UKA. UKA mechanism of failure was documented at the time of surgery and reported in the operative note. The modes of failure were characterized as (1) progression of OA, (2) infection, (3) aseptic femoral component loosening, (4) aseptic tibial component loosening, and (5) other. The “other” category consisted of osteonecrosis, tibial plateau fracture, arthrofibrosis, or unspecified pain. Depending upon the individual case, more than one mechanism of failure may be assigned. Failure was categorized as early failure if revision surgery occurred ≤2 years postoperatively or late failure if revision occurred >2 postoperatively.

The patients were separated into different cohorts based on body mass index (BMI) before their initial procedure. Patients were placed into normal weight (BMI = 20-30 kg/m^2^), obese (BMI ≥ 30.1-40 kg/m^2^), morbidly obese (BMI ≥ 40.1-50 kg/m^2^), and super morbid obese (BMI ≥ 50.1 kg/m^2^) categories (Table 1, Table 2). Each patient received radiographs at every postoperative visit. All radiographs were analyzed for aseptic loosening, polyethylene wear, progression of OA in the contralateral compartment, and alignment. Also, the implant was assessed to discern if there is any overhang that could place stress on the medial collateral ligament, lateral collateral ligament, or anterior/posterior capsule.

**Table 1:** Patient demographics for study participants among the different BMI groups who underwent revision compared to those who did not undergo revision surgery.

Study participants	Revision group (n = 47)	Control group (n = 2178)	*P* value
Age	67.5	68.7	.48
Female	64.5%	67.5%	.82
Medial/lateral UKA (%)	90%/10%	93%/7%	.61
Clinical follow-up (y)	6.3	3.7	.001
Mean BMI (kg/m^2^)	31.8	33.8	.11
Normal BMI (n)	23	853	.35
Obese BMI (n)	18	902	.82
Morbidly obese BMI (n)	4	313	.25
Super morbidly obese BMI (n)	2	110	1.0

**Table 2:** Number (n) and percentages of unicompartmental knee arthroplasty (UKA) revisions per BMI group.

BMI range (kg/m^2^)	Primary UKA (n)	Revision (n)	Revisions (%)
<30	853	23	2.7
30.1-40	902	18	2.0
40.1-50	313	4	1.3
≥50.1	110	2	1.8

### Statistical analysis

A univariate statistical analysis for the data was performed using Student’s t-test for patient demographics, revision rate among BMI groups, and time to revision among BMI groups. The conversion of UKA to TKA revision based on BMI group was analyzed with the chi-square test. For all statistical tests, a *P* value less than 0.05 was defined as statistically significant.

## Results

### Patient profile

A total of 2178 UKA cases were performed. Patient demographics are reported in Table 1. Of note, for both the overall and the revision UKA cohorts, there were consistently higher percentages of female patients than male patients that underwent UKA. The average patient BMI was 33.8 kg/m^2^ (range = 15.5-71.5 kg/m^2^). The median age was 68 years (range = 33-92 years). The mean clinical follow-up period was 3.7 years, and the mean time from index surgery to revision was 7.2 years. Of the 2178 patients, 1167 had a 3-year minimum clinical and radiographic follow-up. We do not have information on the living or deceased status of the patients who did not have 3-year follow-up. There were 853 patients in the normal weight group (BMI = 20-30 kg/m^2^), 902 patients in the obese group (BMI ≥ 30.1-40 kg/m^2^), 313 patients in the morbidly obese group (BMI ≥ 40.1-50 kg/m^2^), and 110 patients in the super morbid obese group (BMI ≥ 50 kg/m^2^).

### Revision rate

**Figure 1:**
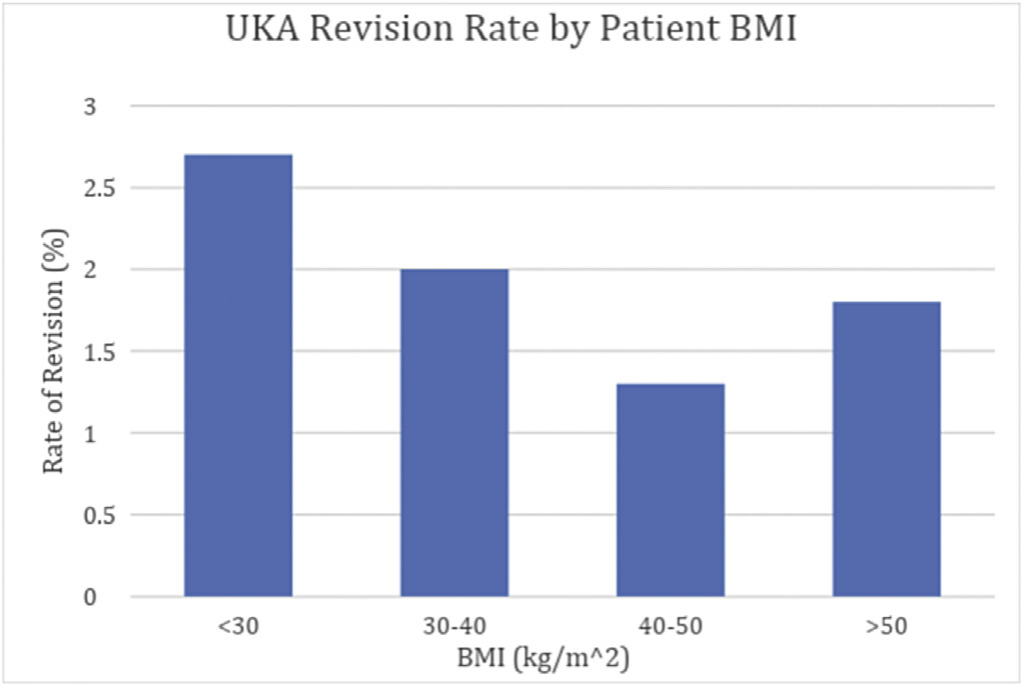
Bar graph illustration of UKA revision rate among all BMI groups.

**Figure 2:**
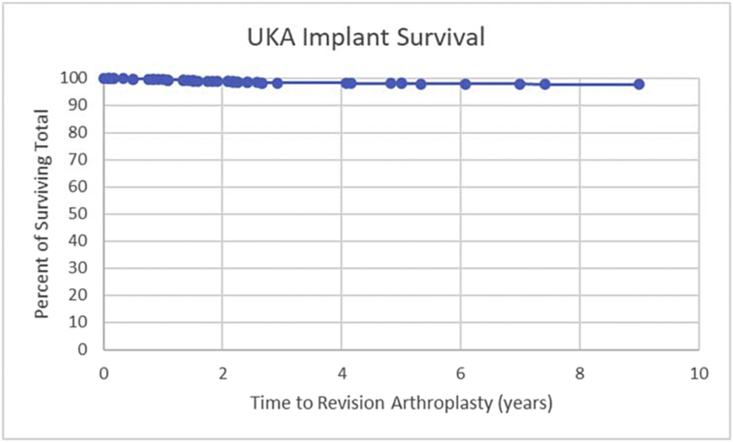
UKA implant survival as a function of time from index surgery.

The revision rate (%) by BMI group for the normal weight group was 2.7% (23/853 patients), obese group was 2.0% (18/902 patients), morbidly obese group 1.3% (4/313 patients), and super morbid obese group 1.8% (2/110 patients), as shown in Table 2. The overall revision rate in this study was 2.2%. No significant difference (*P* < .05) was found for the average BMI of the whole study group (33.8 kg/m^2^) vs the BMI of the revision group (BMI = 32.1 kg/m^2^) (see Table 3). There was a significant difference in the follow-up of the patients undergoing revision as compared against the overall UKA cohort (Table 1). The revision UKA patients were followed up for a longer period secondary to being revised to TKA. There was no significant difference (*P* < .05) among the individual surgeon’s revision rate. No BMI group demonstrated an increased rate of revision to TKA vs the overall population revision rate (Fig. 1). The medial UKA revision rate was 2.6%, and the lateral UKA revision rate was 2.7%. Figure 2 illustrates the implant survivorship observed for this study. There was no statistical significance (*P* < .05) in time to revision among BMI groups (Table 4). The average time to revision was 30.1 months for the normal weight group, 31.5 months for the obese group, 32.6 months for the morbidly obese group, and 43 months for the super morbid obese group.

**Table 3:** Patient BMI statistic data for control and revision groups.

BMI biostatistics	Series (n)	Revision (n)
Group size	2178	47
Mean BMI	33.85	31.75
SD BMI	8.60	7.53
BMI 95% CI Min/Max	33.5-34.2	29.6-33.9

**Table 4:** The time period (mo) from index surgery to revision for UKA for all groups.

BMI (kg/m^2^)	Ave time to revision (mo)	Student’s t-test
20-30	30.17	*P* = .970
30.1-40	31.54	*P* = .920
40.1-50	32.60	*P* = .564
≥50.1	43.0	*P* = .302

### Mechanism of failure

**Figure 3:**
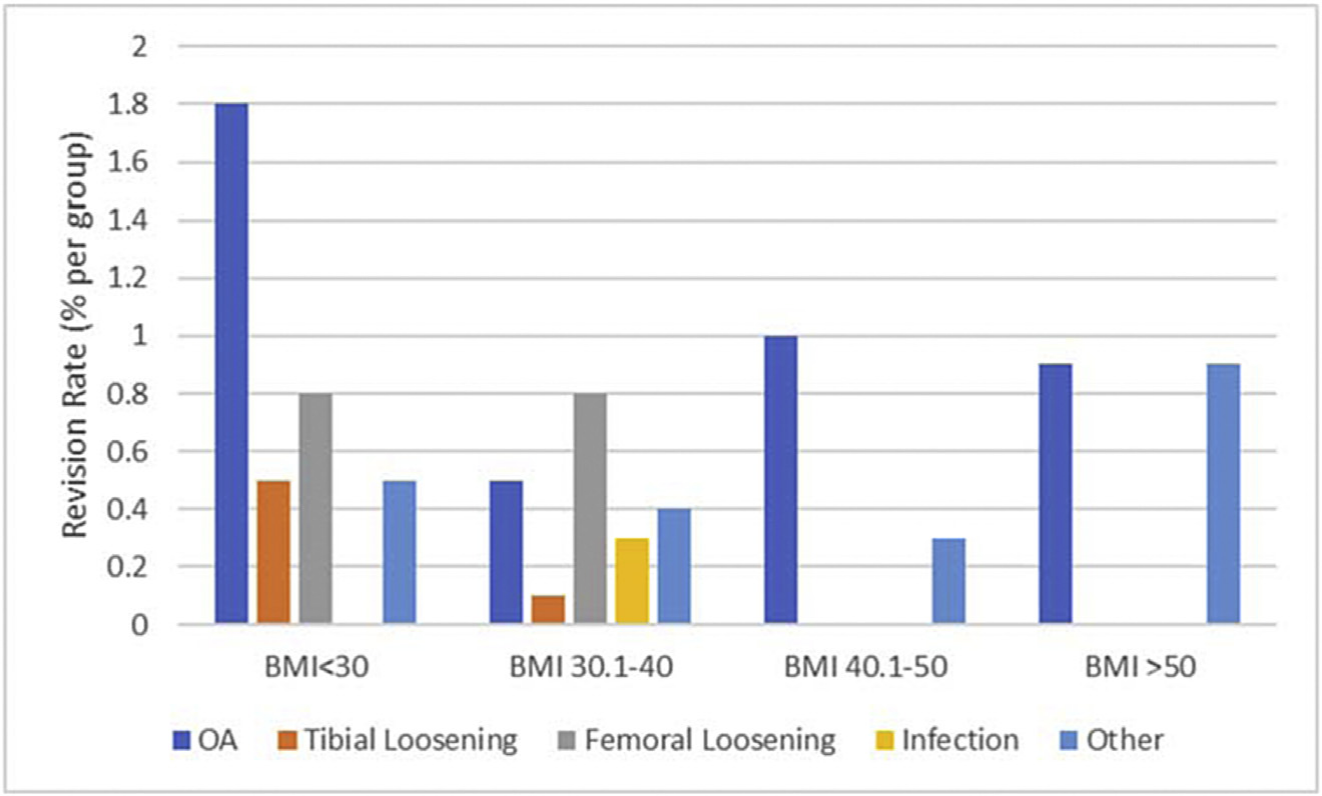
Bar graph illustrating UKA mechanism of failure among all BMI groups. Progression of osteoarthritis was the most common cause of failure among all groups.

Progression of OA was the most common mechanism of failure overall. However, only the normal weight BMI group showed statistical significance (*P* < .05) with progression of OA as a mechanism of failure (*P* = .00038). Aseptic component loosening (femoral or tibial) tended to occur more in normal weight BMI group than in obese BMI groups, but none reached statistical significance. Three infections occurred within the obese BMI group, and all underwent revision (Fig. 3). “Other” failure mechanisms occurred in all BMI groups but were increased in the super morbid BMI (BMI ≥ 50.1 kg/m^2^) (Fig. 3).

**Figure 4:**
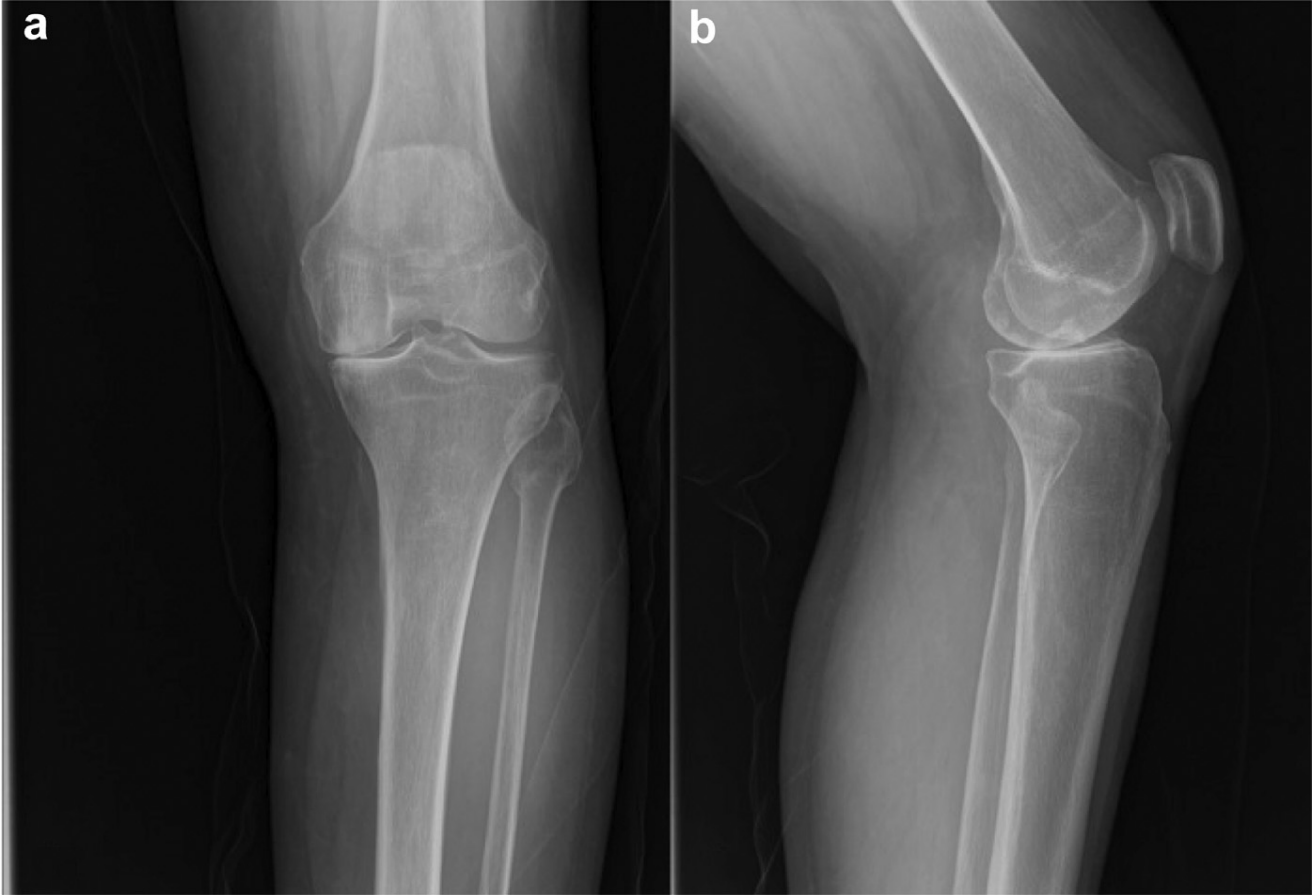
(a) Anteroposterior and (b) lateral radiographs of a study participant showing severe unicompartmental arthritis of the left knee.

(Radiograph courtesy of Mississippi Sports Medicine and Orthopaedic Center).

**Figure 5:**
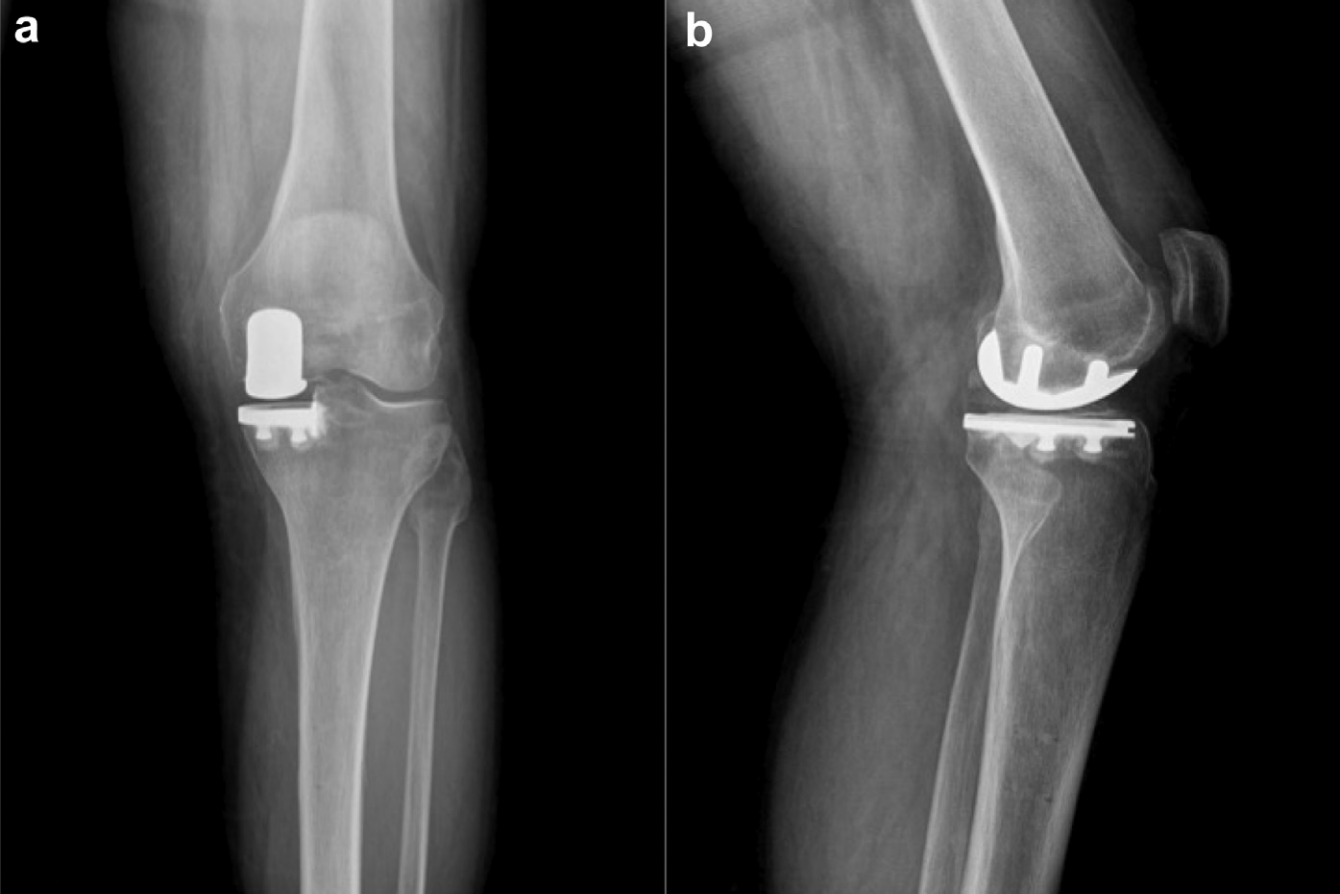
(a) Anteroposterior and (b) lateral radiographs of a study participant after receiving a partial knee replacement at their 8-year follow-up visit.

(Radiographs courtesy of Mississippi Sports Medicine and Orthopaedic Center)

## Discussion

Obesity plays a significant role in the development of knee OA and presents a challenge for surgical treatment [[3], [4], [5], [6], [7], [8],^16^]. The prevalence of OA in the United States is expected to continue rising because of an aging population along with a worsening obesity epidemic [1]. There currently is discordance within the literature to guide the arthroplasty surgeon in regard to UKA in the obese population, and previous dogma has recommended against this procedure in this population [16,17,[24], [25], [26], [27]].

The hallmark article by Kozin and Scott stipulated UKA should not be performed in patients that weigh in excess of 82 kg [16]. To our knowledge, this criterion was based on their clinical experience, and not formally investigated. This weight prerequisite deterred orthopedic surgeons from performing UKA in the obese population. The rational is that obese patients weigh more; UKA involves a smaller surface area that could potentially experience 2-3× load in obese patients. Conversely, there is not any study to our knowledge investigating UKA joint reactive forces in obese patients.

Berend et al. found that performing UKA in patients with a BMI ≥ 32 was associated with an increased rate of implant failure and decreased survivorship [17]. The etiology of failures in this study included deep infection, periprosthetic fracture, aseptic loosening, and persistent pain culminating in conversion to TKA. The sample size of this study was fairly limited with 73 UKAs. Also, there were 2 different prostheses used in the study group [17]. Heck et al. reported that a BMI of ≥32.6 would cause premature failure of UKA [27]. The surgical procedure varied significantly in this investigation; it was a multi-institutional study with patients receiving 2 different prostheses. Nettrour et al. described that UKA in morbidly obese patients was associated with an increased risk of major revision surgery [24]. The sample size analyzing morbidly obese patients was n = 71 patients (89 UKAs). In this study, mobile bearing prostheses were used, and all the mobile bearing spinouts occurred in the morbidly obese group [24].

There are an increasing number of investigations challenging the notion that a BMI ≥ 30 is associated with increased complications. Lum et al. created a 2:1 control group contrasting safety of performing either a UKA or TKA on obese patients [28]. They demonstrated that performing UKA on obese patients was safer than performing TKA [28]. They confirmed that UKA was associated with decreased blood loss, superficial/deep infection, and length of hospital stay compared with TKA in obese patients [28]. Sundaram et al. demonstrated there is not any increase in 30-day perioperative complications in obese patient undergoing UKA [29].

Zengerink et al. described that performing UKA was safe in the obese patients [19]. Obesity did not compromise their implant survivorship at 5 years postoperatively [19]. There study sample included an n = 147 and exclusively focused on medial UKA. Cavaignac et al. published a similar finding that obesity did not affect implant survivorship at 10-year follow-up [30]. Their 10-year survivorship for UKA in obese patients was 92% [30]. But their study group did not specifically focus on the morbidly obese or super morbidly obese patients [30].

The studies by Murray et al. and Molloy et al. are the only ones with a large sample sizes (>900 patients) that have evaluated UKA in obese and morbidly obese patients [31,32]. Both studies reported UKA in obese patients is not associated with decreased survivorship and that increased BMI was associated with a significant improvement in oxford knee scores [31,32]. They both corroborate several smaller sample size study findings that increased BMI does not predispose to implant failure or increased revision rate [18,19,28,30]. To our knowledge, there are no studies specifically investigating the revision rates among the super morbid obese patient (BMI ≥ 50.1 kg/m^2^) population.

In our study, we observed a very low incidence (1.9%) of revision in the morbidly obese (BMI ≥ 40.1-50 kg/m^2^) or super morbidly obese (BMI ≥ 50.1 kg/m^2^) patient populations (n = 423 patients) with a mean follow-up of 76.1 months (≈6.5 years). The revision operations were secondary to aseptic loosening or conversion to TKA due to progression of OA. This is consistent with the most common causes of failure of UKA [33]. There are potential theories as to why we did not see an increased complication or revision rate among obese patient groups. Brainlab helped ensure that during surgery, we did not change the patient’s coronal plane alignment. We understood correcting their mechanical alignment would place stress on the contralateral knee compartments predisposing to progression of OA. It is possible that obese patients are less active than normal weight patients and are not stressing their implants as much. There is a possibility that obese patients may have increased bone strength because of increased reactive forces. The infection rate in the obese group (BMI ≥ 30.1-40 kg/m^2^) was low (0.3%). This is consistent with other studies demonstrating that infections in UKA are not a common cause of UKA failure [21,23].

Our study has several limitations. It is a retrospective study performed at a single institution. This may limit the external validity of our study. We combined both medial and lateral UKA procedures, but the vast majority were medial UKAs (>90%) (Figure 4, Figure 5). Four different fellowship-trained orthopedic surgeons performed the 2178 procedures. However, there was no statistical difference among their individual revision rates. An overall similar technique was used among the 4 surgeons, but there was variability in the UKA volume. However, every patient received the same implant in an attempt to decrease confounding by using multiple prostheses. It is possible a follow-up period of 6 years may not be sufficient to determine implant survivorship. Foran et al. noted failure of TKA implants in their study did not start occurring until 14 years postoperatively [9].

## Conclusions

Increased BMI should not serve as a contraindication to UKA, and contrary to traditional dogma, we found that UKA is a viable option that should be offered to obese patients with unicompartmental end-stage OA. Although the mechanism of UKA failure did vary by patient BMI, the rate of UKA revision was not significantly influenced by patient BMI.

## Conflicts of interest

The authors declare the following financial interests/personal relationships which may be considered as potential competing interests: B. M. Stronach received royalties from Tightline Development, Pacific Research, Signatur Orthopaedics, and MiCare Path; is in the speakers' bureau of DJO Global; is a paid consultant for Smith & Nephew and DJO Global; has stock or stock options in Joint Development LLC and RedCap Cloud; and is a board/committee member of American Association of Hip and Knee Surgeons. T. Pickering is in the speakers' bureau of and a paid consultant for Zimmer Biomet Inc. J. D. Almand is a paid consultant for Zimmer Biomet Inc.

## Appendix A Supplementary data

Conflict of Interest Statement for Almand

Conflict of Interest Statement for Almand

Conflict of Interest Statement for Purcell

Conflict of Interest Statement for Mehrle

Conflict of Interest Statement for Parsell

Conflict of Interest Statement for Pickering

Conflict of Interest Statement for Stronach

Conflict of Interest Statement for Winkler
