# Supplementary material for: Unicompartmental Knee Arthroplasty Is Not Associated With Increased Revision Rates in Obese Patients
Source: Arthroplast Today. 2021 Jun 23;10:12–7. doi: 10.1016/j.artd.2021.05.016 (PMC8242332; doi:10.1016/j.artd.2021.05.016)
Supplement: Conflict of Interest Statement for Almand [file mmc2.pdf]

# INDIVIDUAL CONFLICT OF INTEREST STATEMENT

## *American Association of Hip and Knee Surgeons*

(Adopted from the American Academy of Orthopaedic Surgeons disclosure statement)

The following form **must be filled out completely and submitted by each author (example, 6 authors, 6 forms). All items require a response. If there is no relevant disclosure for a given item, enter "*None.*"**

Unicompartmental Knee Arthroplasty Is Not Associated with Increased Revision Rates in Obese Patients

**Manuscript Title**

1. Royalties from a company or supplier (The following conflicts were disclosed)

   none

2. Speakers bureau/paid presentations for a company or supplier (The following conflicts were disclosed)

   none

3A. Paid employee for a company or supplier (The following conflicts were disclosed)

   none

3B. Paid consultant for a company or supplier (The following conflicts were disclosed)

   none

3C. Unpaid consultants for a company or supplier (The following conflicts were disclosed)

   none

4. Stock or stock options in a company or supplier (The following conflicts were disclosed)

   none

5. Research support from a company or supplier as a Principal Investigator (The following conflicts were disclosed)

   none

6. Other financial or material support from a company or supplier (The following conflicts were disclosed)

   none

7. Royalties, financial or material support from publishers (The following conflicts were disclosed)

   none

8. Medical/Orthopaedic publications editorial/governing board (The following conflicts were disclosed)

   none

9. Board member/committee appointments for a society (The following conflicts were disclosed)

   none

**Each author must sign AND print or type his/her name, date and submit a separate form**

In addition, one BLINDED Conflict of Interest form (no author names used) should be submitted per manuscript with all author disclosures.

| Mariegene Liza Almand | M. Almand | 1/24/21 |
|---|---|---|
| Author Name (Print or Type) | Author Signature | Date |
